# Survey on health, well-being and aging. SABE Colombia 2015: Technical Report

**DOI:** 10.25100/cm.v50i2.4557

**Published:** 2019-06-30

**Authors:** Delia Ortega-Lenis, Fabian Mendez

**Affiliations:** 1 Grupo epidemiología y salud poblacional (GESP). Escuela de Salud Pública. Facultad de Salud. Universidad del Valle. Cali, Colombia; 2 Departamento de Salud Pública y Epidemiología, Pontificia Universidad Javeriana. Cali, Colombia.

**Keywords:** Sample size, aging, health surveys, population surveillance, surveys and questionnaires, healthy aging, elderly, aged, public health surveillance, tamaño de muestra, envejecimiento, encuestas epidemiológicas., vigilancia poblacional, encuesta y cuestionarios, vejez sana, adulto mayor, vejez, vigilancia en salud publica

## Abstract

**Introduction:**

Colombian population is getting old in an accelerated manner, causing economic, social and health services effects. The Ministry of Health and Social Protection in the National System of Population Studies and Surveys for Health implemented the first health, well-being and aging survey- SABE-2015 Colombia- to know the living conditions of people 60 years of age or older.

**Objective:**

Describe the design of the method, statistical sampling and quality control of information from the SABE-2015 survey.

**Methods:**

A cross-sectional study, with quantitative and qualitative approaches, representative for the population in urban and rural areas aged 60 or over. Information was collected on socioeconomic variables, physical and social environment, behavior, cognition and affection, functionality, mental well-being, health conditions, and the use and access to health services.

**Results:**

23,694 surveys were conducted, 17,189 in urban population (72.5%) and 6,505 in rural population. The percentage of effective national response was 66% in 244 municipalities. Supervision was made in 40% of the surveys and telephone re-contact in 25%. The consistency of 100% surveys was reviewed and double entry was developed in 5% of them. National estimates have a 5% margin error.

**Conclusion:**

The SABE Colombia 2015 survey is representative of the main indicators of health, well-being and aging in Colombia. The design allows regional comparisons, between large cities and urban and rural population.

## Resumen

**Introducción:**

La población colombiana está envejeciendo de forma acelerada lo cual está generando efectos económicos, sociales y en los servicios de salud que exigen de información confiable para la planeación y evaluación de políticas en esta población. El Ministerio de Salud y Protección Social implementó la primera encuesta de salud, bienestar y envejecimiento- SABE Colombia 2015- para conocer las condiciones de vida de las personas de 60 años o más.

**Objetivo:**

Describir el diseño del método, del muestreo estadístico y del control de calidad de la información de la encuesta SABE-2015.

**Métodos:**

Encuesta con enfoque cuantitativo y cualitativo, representativo de la población urbana y rural colombiana de 60 años o más. Se registró información socioeconómica, del entorno físico y social, de la conducta, la cognición y el afecto, la funcionalidad, el bienestar mental, las condiciones de salud, y el uso y acceso a servicios de salud.

**Resultados:**

Se realizaron 23,694 encuestas, 17,189 en población urbanas (72.5%) y 6,505 en población rurales. El porcentaje de respuesta efectiva nacional fue del 66% en 244 municipios. Se realizó supervisión en 40% de las encuestas y re-contacto telefónico en 25%. Se revisó la consistencia del 100% de encuestas y doble digitación en el 5%. A nivel nacional las estimaciones tienen un error del 5%.

**Conclusión:**

La encuesta SABE Colombia 2015 es representativa de los principales indicadores de salud, bienestar y envejecimiento en Colombia. El diseño permite hacer comparaciones regionales, entre grandes ciudades y de población urbana y rural.

**Remark**

**Table:** 

**1) Why was this study done?**
To explain the sampling, statistical and quality control design methods applied in the implementation of the SABE Colombia 2015 survey, which support the different analyses generated from the survey.
**2) What did the researchers do and find?**
The percentage of effective national response was 66% in 244 municipalities. The quality assurance of the survey was carried out through an audit and supervision process. Supervision was applied in 40% of the surveys, review of consistency in 100% and with duplication of 5%. National estimates show 5% error.
**3) What do these findings mean?**
This survey provides updated, representative and reliable data on the situation of adults 60 years of age or older at the national, regional level, in major cities and by areas (urban / rural). This information will serve as a basis for different secondary investigations and for the creation of programs and public policies in this population.

## Introduction

The demographic transition is a process that occurs worldwide. Countries move from a state of high fertility, high mortality and a predominance of a young population to a condition characterized by low fertility, low mortality and the preponderance of an old population. The aging process of the world population is happening faster than in the past. In France it went from 7% to 14% in the population proportion of people over 60 in 115 years, in Brazil, Chile, China and India it was estimated that this process will take a little more than 20 years, and in Colombia only 19 years ^1^.

Consequently, population aging has effects on all sectors of society, including health. In addition to the economic effects associated with changes in labor production and consumption related to this stage of the life course, older adults require more support in health services and care, which creates challenges in disease prevention and in the provision of services. It is a priority to know the living conditions of the elderly (defined as a population over 60 years old) to identify higher risk groups, evaluate and adjust public policies and plan services.

Therefore, the Ministry of Health and Social Protection of Colombia, within the National System of Population Studies and Surveys for Health (SNEEPS), set up the development of the Health, Welfare and Aging Survey (SABE-Colombia)^2^. The SABE-Colombia Survey explores several aspects that intervene in the process of aging and during old age stage of urban and rural population, using an interdisciplinary and in-depth approach based on the model of social determinants of health and under the policy framework of the Determinants of Active Aging.

**Figure 1:**
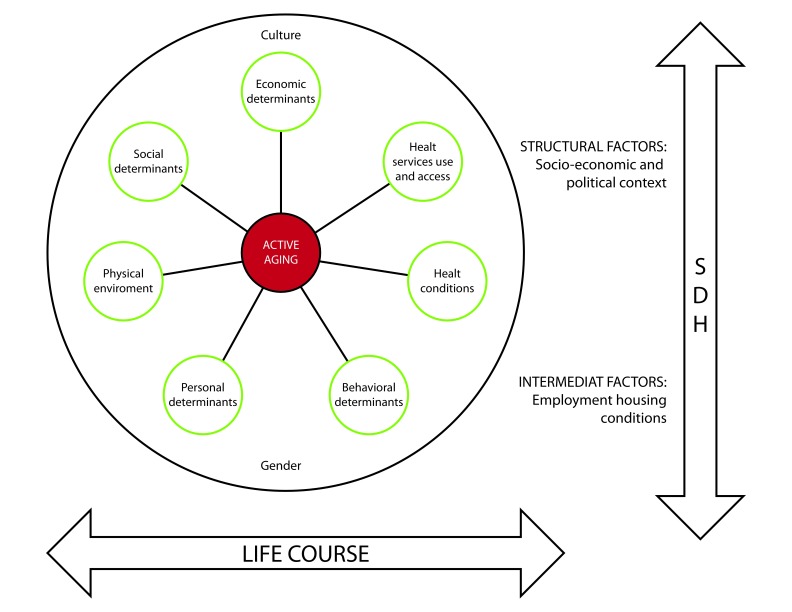
Conceptual model of the SABE Colombia survey. Adapted from the framework of the Determinants of Active aging.

More specifically, from a life course approach, and taking into account the structural and intermediary social determinants that generate health inequities, SABE evaluated different dimensions included in the policy framework of Active Aging, as it is represented in Figure 1. This policy framework was developed by WHO in 2002 ^3^ and is intended to provide useful information, in seven different dimensions, taking into account the socio-cultural context and differences by gender (Figure 1), for the formulation of action plans and for the design of policies that promote healthy aging in nations.

The objective of this article was to describe in detail the methodological design of the survey, including aspects of the statistical sample for the estimation of parameters, the procedures for the collection of information, and the tools for quality assurance and quality control.

## Method and Materials 

### Methodological design

The SABE survey is a cross-sectional study with a quantitative and qualitative methodological approach, representative for the population of men and women 60 years of age or older in the country. In the development of the quantitative component, the study included the application of the following components:

1. A population household survey in urban and rural areas of Colombia, which recorded general information about older adults in relation to socioeconomic variables, physical and social environment, behavior, cognition and affection, functionality, mental well-being, medical and health conditions, and the use and access to health services.

2. Anthropometric measures to all individuals participating in the population survey. These measurements included weight, height, waist circumference, calf circumference, arm circumference, and knee height.

3. Determination of glucose levels, lipid profile and hemoglobin by taking blood samples, in two out of five individuals included in the population survey.

4. Blood pressure measurement in a subsample of older adults participating in the survey.

5. Functionality tests in a subsample of older adults including grip strength, walking speed, balance and time to get up from a chair.

The qualitative component was carried out under an interpretive-comprehensive approach with a focus on symbolic interactionism, which was oriented towards interactions, the dynamics of social activities among people, the meanings attributed to events, the natural environments in which they live, and the actions they perform ^4^.

### Statistical design

The universe of the study was composed of the population residing in urban and rural households in all regions of Colombia (according to the regional divisions of the Ministry of Health and Social Protection); and the target population was all adults 60 years of age and older, non-institutionalized and Spanish speakers. The final sample was composed of those individuals residing in the municipalities randomly selected by region and from cities with a population of over 1.2 million inhabitants (i.e.; “large cities” including Bogotá, Medellin, Cali and Barranquilla). For the sampling frame, the mapping of blocks and units of SNEEPS was used, taking into account the classification by municipalities, urban or rural areas (dispersed population and rural centers).

The geographical coverage of the survey was national, with disaggregation by six major regions: Atlantic, Eastern, Bogotá, Central, Pacific and Orinoquia-Amazonia; large cities and urban-rural stratification (Table 1). The collection period was the year 2015, and the observation units were the individuals.

**Table 1:** Regions and departments that compose the SABE sample.

Regions	Departments
Atlántica	Atlántico, Bolívar, Cesar, Córdoba, La Guajira, Sucre, Magdalena, San Andrés.
Oriental	Norte de Santander, Santander, Boyacá, Cundinamarca, Meta.
Bogotá	Bogotá.
Central	Antioquia, Caquetá, Caldas, Quindío, Risaralda, Tolima, Huila.
Pacífica	Valle, Cauca, Nariño, Chocó.
Orinoquía-Amazonía	Arauca, Casanare, Guainía, Vichada, Vaupés, Amazonas, Guaviare, Putumayo.

### Sample design

The adaptation and adjustment of the sample design for the SABE Colombia Survey was based on the guidelines set forth by SNEEPS, of the Ministry of Health and Social Protection, which considers the implementation and development of a master sample of households for population studies in health ^5^. The sample was probabilistic, cluster, stratified and multi-stage. Each element of the universe had a known and greater than zero probability, the selection of households was made under the grouping of segments (12 dwellings on average); the sample was stratified by urban and rural areas, and the stages were: municipalities (PSU), segments (SSU), housing (HUs) and homes in the fourth stage.

### Sample size

The calculation of the sample was carried out taking into account the regional disaggregation and the forced inclusion of the four large cities. The sample size for these cities, 3,500 individuals per city, was added to the national value by accumulating the sample values for the sub region, region and country. The formulas used corresponded to a simple random sampling, adapted to the cluster design according to Leslie Kish ^6^. The parameters used were a population size of 4,964,793 according to the 2013 DANE projections, a minimum expected proportion of 0.03, a design effect (Deff) of 1.2, a relative standard error (Esrel) of 0.05 and a non-response percentage of 20%. The equation for the estimation of the sample size was as follows:

$$n=\frac{N\;*PQ*Deff}{N*{\left(Esrel*P\right)}^{2}\;+\;(PQ*Deff)}$$

Where *N* is the population size, *P* is the expected minimum prevalence, *Q* is the complement to the prevalence, *Deff* is the design effect due to the conglomeration of units and *Esrel* the relative error. 

According to the previous description, 30,691 surveys were estimated at the national level, 23,162 in the urban area (75.5%) and 7,529 in the rural area (1,908 in populated centers and 5,621 in dispersed rural areas). A total of 6,530 segments, 4,928 urban and 1,602 rural, were planned to obtain the surveys, with an expected average of 4.7 adults per segment.

#### Sample for application of biomarker tests

For this subsample, the calculation of the sample size was carried out taking into account national representation. 86 municipalities were selected, including the 4 large cities. The same formula as for the general sampling was used, assuming an expected proportion of 0.07, a design effect of 1.2, a relative standard error of 0.065 and a non-response percentage of 20%. Obtaining a sample of 4,525 people 60 years of age or older.

The selection of older adults was carried out using systematic sampling, by randomly selecting two out of five individuals of the general sample.

#### Sample for tests of functionality and blood pressure

Based on the same selection of the 86 municipalities for the biomarker subsample, the selection was made for the application of functionality tests and blood pressure assessment in the elderly population. The estimate took into account an expected proportion of approximately 6%, a maximum error of 6% and a non-response percentage of 20%. The sample obtained was 6,161 people with national representation. The selection was done systematically, with a general periodic inclusion of one for every two individuals of the survey.

#### Caregiver samples

For this subsample, convenience sampling was carried out among people residing in households who help to carry out the activities of daily living for adults 60 years of age or older selected in the survey sample. No selection scheme was used; but only those caregivers who agreed to participate in the different municipalities selected in the general sample. The estimated sample was 1,000 people with national representation. 

#### Qualitative study sample

Several sampling strategies were carried out in the qualitative component: geographical, for the convenience of the selection of cities and towns based on ethnographic criteria; and cultural with a classification of the five regions of Colombia; from each region two municipalities were selected for convenience. For the interviews, a sampling of cases of maximum variation was used ^7^, establishing the size across the five regions, two major ethnic groups, two strata in each city or town, two sexes -man and woman-, two age groups -between 60 and 74 years, and 75 and over. The total number of interviews was 14 in each large or intermediate city and 10 in small cities or towns ^8^.

For the focus groups, the call process was established through the sampling of homogeneous cases, with the inclusion criteria as belonging to older adults social groups, a minimum of one year of membership, and diversity in terms of sex and age.

### Estimation process

For the construction of statistical estimators of the parameters of interest and according to the sampling stages, the estimator of the total of the variable was be given by ^9^:

$$\hat{X}=\sum_{h}\sum_{i}F_{hijk}\left(\sum_{j}\sum_{k}x_{hijk}\right)$$

Where: F_hijk_ represents the final expansion factor of the person from the k-th household, the jth USM, the i-th city, the h-th stratum (urban-rural) and X_hijk_ in the same way, the value associated with the characteristic of interest for each adult of 60 years or more observed. The estimation of proportions and reasons were obtained by the following expression:

$$\hat{R}=\frac{\hat{X}}{\hat{Y}}$$

Where each element in the quotient represents a total of one characteristic of interest.

#### Precision calculation

For the evaluation of sampling errors of national estimates, the Last Cluster Method was used ^9^, which states that the greatest contribution to the variance of an estimator in a multi-stage design is that presented between the Primary Sampling Units (PSU). Thus, to obtain the precision of the ratio estimators, the Taylor Series Method was also used.

### Information collection

The information was collected through two processes and consecutively: the segmentation and the application of surveys and subsamples. The segmentation process required a previous review of the cartography delivered and the field survey of the segments for each block. In this process, the fieldworker carried out the recognition and count of the dwellings, for which they took into account the norms established in the manual of lifting and segmentation of the Master Sample of Homes designed for the study in Health, and also identified homes with potential participants for the survey. For the application of the surveys, only the segments allowed by the Ministry of Health for each block were taken.

The SABE survey was applied through a face-to-face interview, using a structured questionnaire with 13 sections: Identification and filter, socioeconomic aspects, physical environment, social environment, behavior, cognition and affection, functionality, medical and health conditions, use and access to health services, anthropometry and functional assessment, link to subsample, subsample record and control data ^10^.

The interviewers visited the homes previously selected in the segmentation process, each carrying elements of study identification. The application of the survey involved the identification of the participants, the registration of demographic data, the signing of informed consent, application of the selection criteria and application of filters through the abbreviated Folstein Mini Mental Test. For older adults, where the score obtained by the test was below 13 points the questions were answered by a companion -proxy-; In this case, the consent of the proxy and the consent of the elderly were diluted.

The anthropometric measurements were taken at the end of the completion of the survey, as well as the functionality tests and blood pressure taking, in the case of having been systematically selected. For the sub-sample of biomarkers, the citation was given to the selected persons, for later taking. For the recording of information, mobile and paper capture devices were used.

All fieldwork personnel were trained for the segmentation process, the application of the survey and the taking of the subsamples. Professionals conducted the training by using strategies such as master classes, individual and group workshops, field activities and sessions to solve questions.

#### Pilot test

The pilot test was carried out in two urban areas (Bogotá and Ubaté) and one rural area (Soledad), located in two regions defined for the collection of the sample. The objective of the test was to evaluate the operationalization of the field work in terms of time, cost, procedures, instruments and adverse events to the different procedures.

This test allowed evaluating the operation of the field survey, the degree of understanding of the questionnaires by the interviewees, the taking of anthropometric measurements, application of functionality tests and biomarkers sampling. On the other hand, the data management process, the capture, the web transmission to the server and the quality control process were also evaluated.

For the biomarker subsample, a pilot test was also carried out in four municipalities in the department of the Valle del Cauca: Cali, Palmira, Candelaria, and Guacarí. This pilot test allowed to review the procedures for a better planning of the field work, specifically related to the selection of the participants, the communication mechanisms, baseline requirements for the sampling, the appointment scheduling process through the call center, transportation and travel scheme, the conditions and measures of biosecurity of technicians and training for the processing of informed consent. Likewise, the formats and manuals of the pre-analytical, sample collection, packaging, transfer and temporary storage of samples based on quality standards were validated.

### Qualitative information

Qualitative data was recorded through a semi-structured interview, observation and focus groups. Each interview followed a guide to ensure obtaining information on the same topics and the same density among participants. All data were recorded and transcribed ^8^.

#### Mechanisms for quality assurance and control

In order to ensure the quality of the data, audit processes and supervision of the survey procedures were designed and implemented. Through the audit, a systematic, independent and documented process of verification of adherence to field staff procedures, information collection procedures, anthropometric measurement protocols, functionality and blood pressure was performed. The audit team was made up of a general coordinator, 5 supervisors and 15 auditors. The audit intervention was effective during the application of the survey ensuring the correct completion of the survey, the informed consent or the processes related to the subsamples.

Likewise, there was another supervisory team in the field, who accompanied the interviewer teams and reported to the regional coordinators, a national coordinator and a central technical area. In this process, the concerns and questions of the segmentation activities, the application of the survey and the subsamples were answered. The monitoring process was developed in 40% of all surveys and telephone or face-to-face contact was made in at least 25% of the surveys.

In parallel, a technical monitoring committee at the central level carried out the monitoring of field work performance. The calculation of indicators was carried out through the information filled out by the supervisors. Some of the most relevant indicators were: housing coverage, percentage of effective response, percentage of rejections, percentage of momentary and permanent absence, number of adults 60 years or older by segment and number of re-visits.

All information resulting from the audit process (checklists and alerts) and supervision was recorded weekly in an online information system with the objective of supporting decision making by supervisors and the coordinator, in addition to feedback processes field work. This system allowed the generation and consultation of the information for the weekly analysis by the technical monitoring committee. For this process, restricted permits were used according to the type of user (supervisor, auditor, coordinator).

On the other hand, the information quality control process had several stages; initially the field supervision process reviewed questionnaire jumps and questions without data, then at the central level a telephone contact was made with 10% of the respondents selected at random, for the corroboration of the data. The subsequent stage was coding and criticism, for which a group of people reviewed the consistency of 100% of the surveys and assigned codes for digitization, in addition the surveys were organized in packages. Finally, in the digitization stage each package was systematized and duplication was performed with 5% of the surveys.

### Data analysis 

The data analysis was carried out taking into account the disaggregation established in the sample design. As they are: national level, urban-rural area and six large regions. For the estimation of the parameters, the expansion factor calibrated according to the population distribution of the DANE 2015 projections was used in the variables: region, sex, age ranges (five-year periods) and urban-rural; this in order to adjust the differences between the distribution of the planned sample and that obtained in the aforementioned variables. It is important to keep in mind that for the study of the variables with prevalence below 0.03 or of the variables different from those mentioned in the adjustment of the factor, the coefficients of variation should be reviewed considering less precise the estimates with coefficients greater than 20% ^11^.

All analyses were performed using the sample design declaration and weighting with the expansion factor in the Stata 14 software ^12^. The tables were obtained using the Tabout command^13^.

This study was approved by the Human Ethics Committee of the Faculty of Health of Universidad del Valle (Acts No. 09-014 and 011-015) and the Bioethics Committee of the Universidad de Caldas (code CBCS-021-14).

## Results

The field work for the sample collection was carried out in 29 weeks. A total of 36,153 older adults were found, of these 23,694 surveys were carried out, for a percentage of national effective response of 66% in 244 municipalities (Table 2). From the total surveys collected, 17,189 were conducted in urban areas (72.5%) and 6,505 in rural areas (1,986 in populated areas and 4,519 in dispersed rural areas). Regarding the estimated sample, 77% of the surveys were carried out. In urban areas the effective response was equal to 62%, 75% in populated areas and 77% in the dispersed rural area (Table 3).

**Table 2:** Response percentage by region.

Region	Effective surveys	Older adults found	% Response
1. Atlántico	6,202	8,608	72
2. Oriental	3,583	5,417	66
3. Orinoquia y Amazonia	1,394	1,806	77
4. Bogotá	2,003	4,557	44
5. Central	6,351	9,583	66
6. Pacífica	4,161	6,182	67
Total	23,694	36,153	66

**Table 3:** Response percentage by region and zone.

	Urban		Populated areas		Rural	
Region	Effective surveys	% response	Effective surveys	% response	Effective surveys	% response
1. Atlántico	4,462	71	757	75	983	77
2. Oriental	2,256	61	222	82	1,105	75
3. Orinoquia y Amazonia	1,327	77	67	87	NA	-
4. Bogotá	1,992	44	NA	-	11	85
5. Central	4,564	64	479	70	1,308	75
6. Pacífica	2,588	62	461	77	1,112	81
Total	17,189	62	1,986	75	4,519	77

 Were worked 8,696 segments, finding a general average of 4.2 adults 60 years of age or older per segment, with variations by region. Regarding the rejections and non-recoverable absences, the general result was 28% and 6% respectively, with differentials by region, where Bogotá presented the highest percentage of rejections (51%). (Table 4).

**Table 4:** Effective segments and Rejection percentage.

Region	Effective segments	Average adults by segment	% rejection	% Non- recoverable Absences
1. Atlántico	2,046	4.2	18	8
2. Oriental	1,309	4.1	26	6
3. Orinoquia y Amazonia	652	2.8	18	4
4. Bogotá	1,238	3.7	51	6
5. Central	1,997	4.8	32	6
6. Pacífica	1,454	4.3	26	3
Total	8,696	4.2	28	6

Regarding the subsamples, in biomarkers there was a final sample of 4,092; for blood pressure, 5,106 measurements; for functionality tests, 4,362 chair tests and 4,831 grip strength; and among caregivers, 1,141 surveys. In the distribution by sex of the general sample there was a higher frequency of women (57.3%), also in the regions, the highest proportion was collected in the Orinoquía and Amazonia (61.2%). By age groups, the highest proportion of surveys were conducted in people between 60 and 64 years (27.7%), in this group 72.7% belonged to the urban area and 58% were women (Table 5.).

**Table 5:** Distribution of the Sample by age group, region, sex and area.

	Residence		Sex		Total
	Urban	Rural	Men	Women	
**Age group**					
60-64	4,772	1,788	2,742	3,818	6,560
65-69	4,014	1,527	2,377	3,164	5,541
70-74	3,089	1,238	1,907	2,420	4,327
75-79	2,448	945	1,477	1,916	3,393
80+	2,866	1,007	1,609	2,264	3,873
**Region**					
Atlántico	4,462	1,740	2,809	3,393	6,202
Oriental	2,256	1,327	1,511	2,072	3,583
Central	4,564	1,787	638	756	1,394
Pacífica	2,588	1,573	777	1,226	2,003
Orinoquía y Amazonía	1,327	67	2,647	3,704	6,351
Bogotá	1,992	11	1,730	2,431	4,161
Total	17,189	6,505	10,112	13,582	23,694

## Discussion

The SABE Colombia survey, maintaining coherence with many of the components of the original SABE, proposed a conceptual framework from which progress was made in the definition of different dimensions of analysis. Consistent with that framework, the results are primarily intended to characterize unfair inequalities in the living conditions of older adults in order to contribute to decision making.

In general, population health surveys are instruments for analyzing the living conditions and well-being of the population that serve to characterize inequities in order to prioritize groups and define or adjust action strategies, programs and public policies.

The results must therefore be reliable and the role of quality control in each phase of the survey protocol is to ensure that the data is obtained in a way that represents the population, that the indicators are free of bias and that the Accuracy of the estimates is necessary to establish comparisons between interest groups. This was the first of the national health surveys to make use of the master sample prepared for the 2013-2023 period, which confers additional advantages to this study within the National Survey System. In addition to reducing the costs of preparation and sampling work, this unique sampling framework follows standardized parameters that favor national representation and geographical subdivisions and will facilitate comparability between different populations studied and with future studies. It is possible that from now on, surveys with panel methodology may also be planned ^2^.

The quality assurance and control processes were carried out permanently and under the supervision and audit of a team of trained professionals during and after the field work. An online information system allowed team members to monitor the work of survey teams and field supervisors, identify early alerts and define actions to follow. In periodic meetings, it was possible to discuss with all stakeholders the progress and difficulties in making decisions about field work.

Despite all the procedures carried out and the rigor that was taken in the various phases of design and development of the Survey, one of the biggest challenges was the achievement of a percentage of response according to the standard values accepted in population sampling. Many surveys worldwide are experiencing increasing percentages of non-response and, although this does not necessarily mean the occurrence of bias or alteration in the estimation of indicators, the most common prescription for the development of population surveys is to minimize non-response ^14^.

There are, however, no single accepted values of percent response in the literature. According to Babbie ^15^ a 50% response percentage is acceptable for analysis and reporting, one of 60% is good and one of 70% or more is very good. Others in contrast suggest that the appropriate minimum response should be 85%. However, several studies by authors cited by Groves ^14^ have led to the impression that non-response is a much smaller threat to survey estimates than previously suggested by practice guides. The SABE survey had a general response rate of 66% (no response of 34%) and also showed consistency with many estimates from other sources that have nationally and internationally measured fundamental parameters of the older adult population. Therefore, we consider that the results of the Survey are a reliable source to characterize this population and contribute appropriately to decision making.

Additionally, the SABE Colombia survey included a qualitative component that complements several of the findings of the quantitative component and contributes to the analysis of the conditions of the elderly population of Colombia through data triangulation approaches.
