# Supplementary material for: Survey on health, well-being and aging. SABE Colombia 2015: Technical Report
Source: Colomb Med (Cali). 2019 Jun 30;50(2):128–38. doi: 10.25100/cm.v50i2.4557 (PMC6774577; doi:10.25100/cm.v50i2.4557)
Supplement: Translation

# Translation: Encuesta de salud, bienestar y envejecimiento sabe Colombia 2015: Reporte técnico

**Contribución del estudio**

**Table:** 

**1)Por que se realizó este estudio?**
Para explicar los métodos del diseño muestral, estadístico y de control de calidad aplicados en la implementación de la encuesta SABE Colombia 2015, los cuales soportan los diferentes análisis generados a partir de la encuesta.
**2) Cuales fueron los resultados relevantes del estudio?**
El porcentaje de respuesta efectiva nacional fue de 66% en 244 municipios. El aseguramiento de calidad de la encuesta se realizó a través de un proceso de auditoría y supervisión. Se aplicó supervisión en 40% de las encuestas, revisión de consistencia en el 100% y doble digitación en 5%. Las estimaciones a nivel nacional presentan errores del 5%.
**3) Que aportan estos resultados?**
Esta encuesta brinda datos actualizados, representativos y confiables sobre la situación de los adultos de 60 años o más a nivel nacional, regional, en ciudades principales y por zonas (urbana/rural). Esta información servirá de base para diferentes investigaciones secundarias y para la creación de programas y políticas públicas en esta población.

## Introducción

La transición demográfica es un proceso que ocurre a nivel mundial. Los países pasan de un estado de alta fertilidad, alta mortalidad y predominio de una población joven a una condición caracterizada por la baja fertilidad, baja mortalidad y la preponderancia de una población vieja. El proceso de envejecimiento de la población mundial está ocurriendo más rápidamente que en el pasado. En Francia pasó del 7% al 14% en la proporción poblacional de personas mayores de 60 años en 115 años, en Brasil, Chile, China y la India se estimó que este proceso tomará un poco más de 20 años y en Colombia en solo 19 años ^1^.

En consecuencia, el envejecimiento poblacional tiene efectos en todos los sectores de la sociedad, incluyendo la salud. Además de los efectos económicos asociados a los cambios en la producción laboral y el consumo relacionados con esta etapa del curso de vida, las personas adultas mayores requieren más soporte en cuidados y en atención en salud lo que genera retos en la prevención de enfermedad y en la provisión de servicios. Es prioritario conocer las condiciones de vida de las personas adultas mayores (definida como población mayor de 60 años) para identificar grupos de mayor riesgo, evaluar y ajustar políticas públicas y planificar servicios.

En consecuencia, el Ministerio de Salud y Protección Social de Colombia estableció, dentro del Sistema Nacional de Estudios y Encuestas Poblacionales para la Salud (SNEEPS), desarrollar la Encuesta de Salud, Bienestar y Envejecimiento (SABE-Colombia) ^2^. La Encuesta SABE explora interdisciplinariamente y en profundidad diversos aspectos que intervienen en el fenómeno del envejecimiento y la vejez de la población colombiana en las áreas urbana y rural desde el modelo de los determinantes sociales de la salud y en el marco de la política de los determinantes del envejecimiento activo.

Más específicamente, desde una aproximación de curso o trayectorias de vida y teniendo en cuenta los determinantes sociales estructurales e intermediarios que generan las reconocidas inequidades en salud, la SABE evaluó las diferentes dimensiones del marco de la política de los determinantes del envejecimiento activo, tal como se representa en la Figura 1. Dicho marco de trabajo para la política fue desarrollado por la OMS en el 2002 ^3^ y tiene el propósito de aportar información útil, en siete dimensiones diferentes teniendo en cuenta el contexto sociocultural y en particular las diferencias de género (Figura 1), para la formulación de planes de acción y para el diseño de políticas que promuevan el envejecimiento saludable en las naciones.

**Figura 1:** Modelo conceptual de la encuesta SABE Colombia. Adaptado desde el marco de los determinantes del envejecimiento activo.

El objetivo de este artículo fue describir en detalle el diseño metodológico de la encuesta, incluyendo aspectos de la muestra estadística para la estimación de parámetros, los procedimientos para la recolección de la información, los mecanismos para aseguramiento y control de calidad.

## Materiales y Métodos

### Diseño metodológico

La encuesta SABE es un estudio de corte transversal, con un enfoque metodológico cuantitativo y cualitativo, representativo para la población de hombres y mujeres de 60 años o más en el país. En el desarrollo del componente cuantitativo de este estudio se incluyó la aplicación de: 

1. Encuesta poblacional en hogares de zonas urbanas y rurales de Colombia, que registró información general de personas adultas mayores en relación con variables socioeconómicas, del entorno físico y social, de la conducta, la cognición y el afecto, la funcionalidad, el bienestar mental, las condiciones médicas y de salud, y el uso y acceso a servicios de salud. 

2. Determinación de los niveles de glucosa, perfil lipídico y hemoglobina mediante la toma de muestras de sangre, realizadas en dos de cada cinco personas incluidas en la encuesta poblacional. 

3.Toma de medidas antropométricas a la totalidad de las personas adultas mayores participantes en la encuesta poblacional. Estas medidas incluyen peso, talla, circunferencia de cintura, circunferencia de pantorrilla, circunferencia del brazo y la altura de rodilla. 

4.Submuestra de presión arterial, aplicada de manera efectiva a las personas adultas mayores del país, participantes en la encuesta. 

5. Submuestra de pruebas de funcionalidad como fuerza de agarre, velocidad de la marcha, equilibrio y tiempo de incorporarse de una silla. 

El componente cualitativo se realizó bajo una mirada interpretativa-comprensiva con un enfoque de interaccionismo simbólico, orientado a las interacciones, la dinámica de las actividades sociales entre las personas, los significados que atribuyen a los eventos, los ambientes naturales en que viven y las acciones que desempeñan ^4^.

### Diseño estadístico

El universo del estudio estuvo compuesto por la población residentes en hogares urbanos y rurales de todas las regiones de Colombia (de acuerdo a la conformación del Ministerio de Salud y Protección Social); La población objetivo fueron todos los adultos de 60 años y más, no institucionalizados, de habla hispana y finalmente la muestra estuvo compuesta por aquellos adultos mayores residentes en los municipios seleccionados aleatoriamente por cada región y de las grandes ciudades (Bogotá, Medellín, Cali y Barranquilla) con población superior a 1.2 millones de habitantes. Para el marco muestral se utilizó la cartografía de manzanas y unidades del SNEEPS, teniendo en cuenta la clasificación por municipios, zonas urbanas o rurales (centro poblado y rural disperso).

La cobertura geográfica de la encuesta fue nacional, con desagregación de seis grandes regiones como Atlántica, Oriental, Bogotá, Central, Pacífica y Orinoquía-Amazonía, grandes ciudades y estratificación urbano-rural (Tabla 1). El periodo de recolección fue el año 2015. Por último, las unidades de observación fueron cada uno de los adultos mayores de 60 años. 

**Tabla 1:** Regiones y departamentos que conforman la muestra SABE.

Región	Departamentos
Atlántica	Atlántico, Bolívar, Cesar, Córdoba, La Guajira, Sucre, Magdalena, San Andrés.
Oriental	Norte de Santander, Santander, Boyacá, Cundinamarca, Meta.
Bogotá	Bogotá.
Central	Antioquia, Caquetá, Caldas, Quindío, Risaralda, Tolima, Huila.
Pacífica	Valle, Cauca, Nariño, Chocó.
Orinoquía-Amazonía	Arauca, Casanare, Guainía, Vichada, Vaupés, Amazonas, Guaviare, Putumayo.

### Diseño muestral

La adaptación y ajuste del diseño muestral para la Encuesta SABE Colombia se basó en los lineamientos dispuestos por el SNEEPS, del Ministerio de Salud y Protección social, que considera la implementación y desarrollo de una muestra maestra de hogares para estudios poblacionales en salud ^5^. La muestra fue probabilística, de conglomerados, estratificada y polietápica. Cada elemento del universo tuvo una probabilidad conocida y mayor a cero, la selección de los hogares se realizó bajo la agrupación de segmentos (12 viviendas en promedio); la muestra fue estratificada por zona urbana y rural, y las etapas fueron: municipios (UPM), segmentos (USM), viviendas (UTM) y hogares en la cuarta etapa.

### Tamaño de muestra

El cálculo de la muestra se realizó teniendo en cuenta la desagregación regional y la inclusión forzosa de las cuatro grandes ciudades. El tamaño de la muestra para estas ciudades se adicionó al valor nacional al acumular los valores muestrales para subregión, región y país, este se fijó en 3,500 adultos mayores en cada ciudad. Las fórmulas utilizadas correspondieron a un muestreo aleatorio simple, adaptadas al diseño de conglomerados de acuerdo con Leslie Kish ^6^. Los parámetros utilizados fueron un tamaño poblacional de 4,964,793 de acuerdo con la proyección DANE 2013, una proporción mínima esperada de 0.03, efecto de diseño (Deff) de 1.2, error estándar relativo (Esrel) de 0.05 y un porcentaje de no respuesta del 20%. La ecuación para la estimación del tamaño de muestra fue la siguiente:

$$n=\frac{N\;*PQ*Deff}{N*{\left(Esrel*P\right)}^{2}\;+\;(PQ*Deff)}$$

Donde *N* es el tamaño poblacional, *P* es la prevalencia mínima esperada, *Q* es el complemento de la prevalencia, *Deff* es el efecto de diseño debido a la conglomeración de unidades y *Esrel* el error relativo. 

De acuerdo con el esquema anterior se obtuvieron 30,691 encuestas esperadas a nivel nacional, 23,162 en la zona urbana (75.5%) y 7,529 en la zona rural (1,908 en centros poblados y 5,621 en rural disperso). Se planearon 6,530 segmentos, 4,928 urbanos y 1,602 rurales para la obtención de las encuestas, teniendo en cuenta un promedio de 4.7 adultos por segmento.

#### Muestra para la aplicación de pruebas de biomarcadores

Para esta submuestra el cálculo del tamaño muestral se realizó teniendo en cuenta representatividad nacional. Se seleccionaron 86 municipios, incluyendo las 4 grandes ciudades. Se utilizó la misma fórmula de la muestra general, asumiendo una proporción esperada de 0.07, un efecto de diseño de 1.2, un error estándar relativo de 0.065 y un porcentaje de no respuesta del 20%. Obteniendo una muestra de 4,525 personas de 60 años o más.

La selección de los adultos mayores se realizó de forma sistemática, calculando un salto muestral de acuerdo a la muestra general en cada municipio seleccionado, en este caso, dos de cada cinco personas.

#### Muestra para pruebas de funcionalidad y presión arterial

Con base en la misma selección de los 86 municipios para la submuestra de biomarcadores, se realizó la selección para la aplicación de pruebas de funcionalidad y valoración de la presión arterial en la población adulta mayor. La estimación tuvo en cuenta una proporción esperada aproximada de 6%, un error máximo de 6% y un porcentaje de no respuesta de 20%. La muestra obtenida fue de 6,161 personas con representatividad nacional. La selección se realizó de forma sistemática, con un salto general de uno por cada dos adultos mayores de 60 años de la encuesta. 

#### Muestra cuidadores

Para esta submuestra se realizó un muestreo a conveniencia sobre personas residentes en hogares que ayudan a realizar las actividades de la vida diaria a los adultos de 60 años o más seleccionados en la muestra de la encuesta. No se utilizó esquema de selección, sólo aquellos cuidadores que aceptaran participar en los diferentes municipios seleccionados en la muestra general. La muestra estimada fue de 1,000 personas con representatividad nacional.

#### Muestra estudio cualitativo

Se llevaron a cabo varios tipos de muestreo: geográfico, por conveniencia para la selección de ciudades y pueblos basados en el criterio etnográfico, y cultural con una clasificación de las cinco regiones de Colombia; de cada una se seleccionaron dos municipios por conveniencia. Para las entrevistas se utilizó un muestreo de casos de variación máxima ^7^, estableciendo el tamaño a través de las cinco regiones, dos etnias mayoritarias, dos estratos en cada ciudad o pueblo, dos sexos -hombre y mujer-, dos grupos de edad -entre 60 y 74 años, y 75 y más-. El total de entrevistas realizadas fue 14 en cada ciudad grande o intermedia y 10 en ciudades pequeñas o pueblos ^8^.

Para los grupos focales, el proceso de convocatoria se estableció a través del muestreo de casos homogéneos teniendo como criterios de inclusión pertenecer a grupos de socialización de adultos mayores, con mínimo un año de pertenencia al mismo, diverso en cuanto a sexo y edad.

### Procesos de estimación

Para la construcción de estimadores estadísticos de los parámetros de interés y conforme las etapas de muestreo, el estimador del total de la variable estará dado por^9^:

$$\hat{X}=\sum_{h}\sum_{i}F_{hijk}\left(\sum_{j}\sum_{k}x_{hijk}\right)$$

Donde: F_hijk_ representa el factor final de expansión de la persona del k-ésimo hogar, de la j-ésima USM, de la i-ésima ciudad, del h-ésimo estrato (urbano-rural) y X_hijk_ de la misma forma, el valor asociado a la característica de interés para cada adulto de 60 años o más observado. La estimación de proporciones y razones se obtienen mediante la siguiente expresión:

$$\hat{R}=\frac{\hat{X}}{\hat{Y}}$$

Donde cada elemento en el cociente representa un total de una característica de interés.

#### Cálculo de precisión

Para la evaluación de los errores de muestreo de las estimaciones nacionales se utilizó el método de Conglomerados Últimos ^9^, el cual plantea que la mayor contribución a la varianza de un estimador en un diseño de múltiples etapas es la que se presenta entre las unidades primarias de muestreo (UPM). De esta forma, para obtener las precisiones de los estimadores de razón, se usó además el método de series de Taylor.

### Recolección de Información

La recolección de la información se realizó a través de dos procesos y de forma consecutiva: la segmentación y la aplicación de encuestas y submuestras. El proceso de segmentación requirió una revisión previa de la cartografía entregada y el levantamiento en campo de los segmentos por cada manzana. En este proceso el segmentador realizó el reconocimiento de las viviendas y su recuento, para la cual tuvo en cuenta las normas establecidas en el manual de levantamiento y segmentación de la Muestra Maestra de Hogares diseñado para el estudio en Salud, además se incluyó la identificación de las viviendas con potenciales participantes para la encuesta. Para la aplicación de las encuestas sólo se tomaron los segmentos permitidos por el Ministerio de Salud por cada manzana.

La encuesta SABE se aplicó a través de una entrevista cara a cara, utilizando un cuestionario estructurado con 13 secciones: Identificación y filtro, aspectos socioeconómicos, medio ambiente físico, medio ambiente social, conducta, cognición y afecto, funcionalidad, condiciones médicas y de salud, uso y acceso a servicios de salud, antropometría y valoración funcional, enlace a submuestra. registro submuestra y datos de control ^10^.

Los encuestadores visitaron los hogares seleccionados previamente en el proceso de segmentación, portando cada uno elementos de identificación del estudio. La aplicación de la encuesta implicó la identificación de las personas participantes, el registro de los datos demográficos, la firma del consentimiento informado, aplicación de los criterios de selección y aplicación de los filtros a través del Minimental Test de Folstein abreviado. Para los adultos mayores, donde el puntaje obtenido por el test estuvo por debajo de 13 puntos las preguntas fueron respondidas por un acompañante -proxy-; en este caso se diligenció el consentimiento del proxy y el asentimiento del adulto mayor.

La toma de las medidas antropométricas se realizó al final del diligenciamiento de la encuesta, así como las pruebas de funcionalidad y la toma de la presión arterial, en el caso de haber sido seleccionado sistemáticamente. Para la submuestra de biomarcadores se entregó la citación a las personas seleccionadas, para su posterior toma. Para el registro de la información se utilizaron dispositivos móviles de captura y en papel.

Todo el personal de trabajo de campo fue capacitado para el proceso de segmentación, la aplicación de la encuesta y la toma de las submuestras. El entrenamiento fue realizado por profesionales utilizando estrategias como clases magistrales, talleres individuales y grupales, actividades en campo y sesión de preguntas.

#### Prueba piloto

La prueba piloto se realizó en dos zonas urbanas (Bogotá y Ubaté) y una zona rural (Soledad), ubicadas en dos regiones definidas para la recolección de la muestra. El objetivo de la prueba fue evaluar la operativización del trabajo de campo en términos de tiempo, costo, procedimientos, instrumentos y eventos adversos a los diferentes procesos.

Esta prueba permitió evaluar el funcionamiento de la encuesta en campo, el grado de comprensión de los cuestionarios por parte de los entrevistados, la toma de mediciones antropométricas, de biomarcadores y las pruebas de funcionalidad. Por otro lado, también fueron evaluados los procesos de administración de datos, la captura, la transmisión vía web al servidor y el proceso de control de calidad. 

Para la submuestra de biomarcadores también se realizó una prueba piloto en cuatro municipios del departamento del valle del cauca: Cali, Palmira, Candelaria, y Guacarí. Esta prueba piloto permitió revisar los procedimientos para una mejor planeación del trabajo de campo, específicamente lo relacionado con la selección de los participantes, los mecanismos de comunicación, requisitos basales para la toma de la muestra, el proceso de programación de citas a través de la central de llamadas, esquema de transporte y desplazamiento, las condiciones y medidas de bioseguridad de los técnicos y el entrenamiento para el diligenciamiento del consentimiento informado. Así mismo, se validaron los formatos y manuales de las etapas de preanalítica, de toma de las muestras, embalaje, traslado y el almacenamiento temporal de las muestras con base en los estándares de calidad.

#### Información cualitativa

El registro de datos cualitativos se realizó a través de una entrevista semiestructurada, la observación y el grupo focal. Cada entrevista siguió una guía para garantizar la obtención de información sobre los mismos temas y de la misma densidad entre participantes. Todos los datos fueron grabados y transcritos textualmente ^8^.

### Mecanismos para el aseguramiento y control de calidad

Para el aseguramiento de la calidad de los datos se diseñaron e implementaron procesos de auditoría y supervisión de los procedimientos de la encuesta. A través de la auditoría se realizó un proceso sistemático, independiente y documentado de verificación de la adherencia a los procedimientos del personal de campo, procedimientos de recolección de información, protocolos de toma de medidas antropométricas, de funcionalidad y presión arterial. El equipo de auditoría estuvo conformado por un coordinador general, 5 supervisores y 15 auditores. La intervención de la auditoria se hizo efectiva durante la aplicación de la encuesta asegurando el correcto diligenciamiento de la encuesta, el consentimiento informado o de los procesos relacionados con las submuestras.

Así mismo, se contó con otro equipo de supervisión en campo, los cuales acompañaron a los equipos de encuestadores y reportaron a los coordinadores regionales, a un coordinador nacional y a una área técnica central. En este proceso se dió respuesta a las inquietudes y las preguntas de las actividades de segmentación, a la aplicación de la encuesta y de las submuestras. El proceso de supervisión se desarrolló en el 40% de todas las encuestas y se realizó re-contacto telefónico o presencial en al menos el 25% de las encuestas.

De forma paralela, se llevó a cabo el seguimiento al desempeño del trabajo de campo a nivel central, por parte del comité técnico de seguimiento. Se realizó el cálculo de indicadores a través de la información diligenciada por parte de los supervisores. Algunos de los indicadores más relevantes fueron: cobertura de viviendas, porcentaje de respuesta efectiva, porcentaje de rechazos, porcentaje de ausencia momentáneas y permanentes, número de adultos de 60 años o más por segmento y número de re-visitas.

Toda la información producto del proceso de auditoría (listas de chequeo y alertas) y supervisión se registró semanalmente en un sistema de información en línea con el objetivo de apoyar la toma de decisiones por parte de los supervisores y del coordinador, además retroalimentar los procesos del trabajo del campo. Este sistema permitió la generación y consulta de la información para el análisis semanal por parte del comité técnico de seguimiento. Para este proceso se utilizaron permisos restringidos de acuerdo con el tipo de usuario (supervisor, auditor, coordinador).

Por otro lado, el proceso del control de la calidad de la información contó con varias etapas; inicialmente el proceso de supervisión en campo revisaba saltos y preguntas sin dato, luego al nivel central se realizó un contacto telefónico con el 10% de los encuestados seleccionadas al azar, para la corroboración de los datos. La etapa posterior fue la codificación y crítica, para la cual un grupo de personas revisaron la consistencia del 100% de las encuestas y asignaron códigos para la digitalización, además se organizaron las encuesas en paquetes. Finalmente, en la etapa de digitalización cada paquete fue sistematizado y se realizó doble digitación con el 5% de las encuestas.

### Análisis de datos

El análisis de los datos se realizó teniendo en cuenta las desagregaciones establecidas en el diseño muestral. Como son: nivel nacional, zona urbano-rural y seis grandes regiones. Para la estimación de los parámetros se utilizó el factor de expansión calibrado de acuerdo con la distribución poblacional de las proyecciones del DANE 2015 en las variables: región, sexo, rangos de edad (quinquenios) y urbano-rural; Esto con el objetivo de ajustar las diferencias entre la distribución de la muestra planeada y la obtenida en las variables antes mencionadas. Es importante tener en cuenta que para el estudio de las variables con prevalencias por debajo de 0.03 o de las variables diferentes a las mencionadas en el ajuste del factor, se deben revisar los coeficientes de variación considerando las estimaciones con coeficientes mayores al 20% como poco precisas ^11^.

Todos los análisis se realizaron utilizando la declaración del diseño muestral y la ponderación con el factor de expansión en el software Stata 14 ^12^. Las tablas fueron obtenidas utilizando el comando tabout ^13^.

Este estudio contó con el aval del comité de ética Humana de la facultad de Salud de la Universidad del Valle (actas No. 09-014 y 011-015) y del comité de Bioética de la Universidad de Caldas (código CBCS-021-14).

## Resultados

El trabajo de campo para la recolección de la muestra se realizó en 29 semanas. Se encontraron 36,153 personas adultas mayores, de estas se lograron realizar 23,694 encuestas, para un porcentaje de respuesta efectiva nacional del 66% en 244 municipios (Tabla 2). Del total de encuestas recolectadas, 17,189 se realizaron en las zonas urbanas (72.5%) y 6,505 en las zonas rurales (1,986 en centros poblados y 4,519 en zonas rurales dispersas). Respecto a la muestra estimada, se realizó el 77% de las encuestas. En las zonas urbanas la respuesta efectiva fue igual al 62%, 75% en los centros poblados y del 77% en la zona rural dispersa (Tabla 3 ).

**Tabla 2:** Porcentaje de respuesta por región.

Regionales	Encuestas efectivas	Adultos encontrados	% Respuesta
1. Atlántico	6,202	8,608	72
2. Oriental	3,583	5,417	66
3. Orinoquia y Amazonia	1,394	1,806	77
4. Bogotá	2,003	4,557	44
5. Central	6,351	9,583	66
6. Pacífica	4,161	6,182	67
**Total general**	**23,694**	**36,153**	**66**

**Tabla 3:** Porcentaje de respuesta por región y zona.

	Urbano		Centro poblado		Rural	
Regionales	encuestas efectivas	% respuesta	encuestas efectivas	% respuesta	encuestas efectivas	% respuesta
1. Atlántico	4,462	71	757	75%	983	77
2. Oriental	2,256	61	222	82%	1,105	75
3. Orinoquia y Amazonia	1,327	77	67	87%	NA	-
4. Bogotá	1,992	44	NA	-	11	85
5. Central	4,564	64	479	70%	1,308	75
6. Pacífica	2,588	62	461	77%	1,112	81
Total general	17,189	62	1,986	75%	4,519	77

Se trabajaron 8,696 segmentos, encontrando un promedio general de 4.2 personas adultas de 60 años o más por segmento, con variaciones por región. Respecto a los rechazos y ausencias no recuperables, el resultado general fue del 28% y 6% respectivamente, con diferenciales por región, donde Bogotá presentó el porcentaje más alto de rechazos (51%). (Tabla 4)

**Tabla 4:** Segmentos efectivos y porcentaje de rechazos.

Regional	Total segmentos efectivos	Promedio de personas por segmento	% rechazos	% Ausentes no recuperables
1. Atlántico	2,046	4.2	18	8
2. Oriental	1,309	4.1	26	6
3. Orinoquia y Amazonia	652	2.8	18	4
4. Bogotá	1,238	3.7	51	6
5. Central	1,997	4.8	32	6
6. Pacífica	1,454	4.3	26	3
**Total general**	**8,696**	**4.2**	**28**	**6**

Respecto a las submuestras, biomarcadores se tuvo una muestra final de 4,092, presión arterial 5,106, pruebas de funcionalidad (silla: 4,362, fuerza de agarre: 4,831) y cuidador con 1,141 encuestas. En la distribución por sexo de la muestra general se presentó mayor frecuencia de mujeres (57.3%), así mismo en las regiones, la mayor proporción se recolectó en la Orinoquía y Amazonía (61.2%). Por grupos de edad, la mayor proporción de encuestas se realizó en personas entre 60 y 64 años (27.7%), en este grupo 72.7% perteneciann a la zona urbana y 58% fueron mujeres (Tabla 5.).

**Tabla 5:** Distribución de la Muestra por grupo de edad, región, sexo y zona.

	Residencia		Sexo		Total
	Urbana	Rural	Hombre	Mujer	
Grupo Edad					
60-64	4,772	1,788	2,742	3,818	6,560
65-69	4,014	1,527	2,377	3,164	5,541
70-74	3,089	1,238	1,907	2,420	4,327
75-79	2,448	945	1,477	1,916	3,393
80+	2,866	1,007	1,609	2,264	3,873
**Región**					
Atlántico	4,462	1,740	2,809	3,393	6,202
Oriental	2,256	1,327	1,511	2,072	3,583
Central	4,564	1,787	638	756	1,394
Pacífica	2,588	1,573	777	1,226	2,003
Orinoquía y Amazonía	1,327	67	2,647	3,704	6,351
Bogotá	1,992	11	1,730	2,431	4,161
**Total**	17,189	6,505	10,112	13,582	23,694

## Discusión

La encuesta SABE Colombia, manteniendo coherencia con muchos de los componentes de la SABE original, propuso un marco conceptual desde el que se avanzó en la definición de diferentes dimensiones de análisis. En coherencia con ese marco, los resultados tienen como principal propósito caracterizar las desigualdades injustas en las condiciones de vida de las personas adultas mayores con el fin de aportar a la toma de decisiones. 

En general, las encuestas poblacionales en salud son instrumentos de análisis de las condiciones de vida y bienestar de la población que sirven para caracterizar las inequidades con el propósito de priorizar grupos y definir o ajustar estrategias de acción, programas y políticas públicas.

Los resultados deben por tanto ser confiables y el papel del control de calidad en cada una de las fases del protocolo de la encuesta es asegurar que los datos sean obtenidos de manera que representen a la población, que los indicadores estén libres de sesgos y que la precisión de las estimaciones sea la necesaria para establecer comparaciones entre grupos de interés. 

Fue esta la primera de las encuestas nacionales en salud en hacer uso de la muestra maestra elaborada para el periodo 2013-2023, lo cual confiere ventajas adicionales a este estudio dentro del Sistema Nacional de Encuestas. Además de la reducción de costos de preparación y de trabajo de muestreo, este marco muestral único sigue unos parámetros estandarizados que favorecen la representatividad nacional y por subdivisiones geográficas y facilitarán la comparabilidad entre diferentes poblaciones estudiadas y con futuros estudios. Es posible que en adelante puedan planificarse además encuestas con metodología panel ^2^.

Los procesos de aseguramiento y control de calidad se llevaron a cabo de manera permanente y bajo la supervisión y auditoría de un equipo de profesionales entrenados durante y después del trabajo de campo. Un sistema de información en línea permitía a los miembros del equipo hacer seguimiento al trabajo de los equipos de encuestadores y supervisores en campo, identificar tempranamente alertas y definir acciones a seguir. En reuniones periódicas se pudieron discutir con todos los interesados los avances y dificultades para la toma de decisiones sobre el trabajo de campo.

A pesar de todos los procedimientos llevados a cabo y de la rigurosidad que se tuvo en las diversas fases de diseño y desarrollo de la Encuesta, uno de los mayores retos fue el logro de un porcentaje de respuesta acorde con los valores estándar aceptados en muestreos poblacionales. Muchas encuestas a nivel mundial están experimentando porcentajes de no respuesta crecientes y, aunque esto no necesariamente significa la ocurrencia de sesgo ni alteración en la estimación de los indicadores, la prescripción más común para el desarrollo de encuestas poblacionales es minimizar la no respuesta ^14^.

No hay sin embargo valores únicos aceptados de porcentaje de respuesta en la literatura. De acuerdo a Babbie ^15^ un porcentaje de respuesta de 50% es aceptable para realizar análisis y reportar, uno de 60% es bueno y uno de 70% o más es muy bueno. Otros en contraste sugieren que la respuesta mínima adecuada debería ser 85%. No obstante, varios estudios de autores citados por Groves ^14^ han llevado a la impresión de que la no respuesta es una amenaza mucho más pequeña para las estimaciones de las encuestas que lo sugerido anteriormente por las guías de práctica. La encuesta SABE tuvo un porcentaje general de respuesta de 66% (no respuesta de 34%) y además mostró consistencia con muchas estimaciones de otras fuentes que han medido nacional e internacionalmente parámetros fundamentales de la población adulta mayor. Por lo anterior, consideramos que los resultados de la Encuesta son fuente confiable para caracterizar esta población y contribuir de manera apropiada a la toma de decisiones.

Adicionalmente, la encuesta SABE Colombia incluyó un componente cualitativo que complementa varios de los hallazgos del componente cuantitativo y aporta al análisis de las condiciones de la población adulta mayor de Colombia mediante aproximaciones de triangulación de datos.
